# Quantifying trade-offs between ecological gains, economic costs, and landowners’ preferences in boreal mire protection

**DOI:** 10.1007/s13280-021-01530-0

**Published:** 2021-04-06

**Authors:** Eini Nieminen, Santtu Kareksela, Panu Halme, Janne Sakari Kotiaho

**Affiliations:** 1 Department of Biological and Environmental Science, University of Jyväskylä, P.O. Box 35, 40014 Jyväskylä, Finland; 2 Parks and Wildlife Finland, Metsähallitus, P.O. Box 36, 40101 Jyväskylä, Finland

**Keywords:** Conservation policy, Private land protection, Systematic conservation planning, Trade-off analysis, Voluntary conservation, Zonation

## Abstract

Private land often encompasses biodiversity features of high conservation value, but its protection is not straightforward. Commonly, landowners’ perspectives are rightfully allowed to influence conservation actions. This unlikely comes without consequences on biodiversity or other aspects such as economic considerations, but these consequences are rarely quantitatively considered in decision-making. In the context of boreal mire protection in Finland, we report how acknowledging landowners’ resistance to protection changes the combination of mires selected to conservation compared to ignoring landowners’ opinions. Using spatial prioritization, we quantify trade-offs arising between the amount of landowners’ resistance, protected biodiversity, and financial costs in different conservation scenarios. Results show that the trade-offs cannot be fully avoided. Nevertheless, we show that the systematic examination of the trade-offs opens up options to alleviate them. This can promote the evaluation of different conservation policy outcomes, enabling better-informed conservation decisions and more effective and socially sustainable allocation of conservation resources.

**Supplementary Information:**

The online version contains supplementary material available at 10.1007/s13280-021-01530-0.

## Introduction

In the face of global environmental threats such as climate change (IPCC 2018) and biodiversity decline (Dirzo et al. 2014), wetlands contribute remarkably to the wellbeing of nature and human. They provide globally significant ecosystem services such as climate regulation, water regulation and purification, and recreation (Zedler and Kercher 2005; de Groot et al. 2012). In addition to their high economic, social, and cultural values (e.g.,de Groot et al. 2012; Patton et al. 2015; Papayannis and Pritchard 2018), wetlands serve as habitats for vast number of species of many taxa (Junk et al. 2006).

Peatlands are wetlands covering approximately 4–4.6 million km^2^ or ca. 3% of global land area (Parish et al. 2008). Over 80% of them are located on boreal areas (Yu et al. 2010). If protected and restored, peatlands have a high potentiality in mitigating climate change as their capacity to store carbon is considerable in relation to their area: globally, they store twice as much carbon as the forest biomass (Leifeld and Menichetti 2018). When wetlands in general are ecosystems characterized by the presence of water (Ramsar 1971), peatlands’ biodiversity and their function as ecosystems depend specifically on a water Table level (e.g.,Laine et al. 1995; Haapalehto et al. 2011; Maanavilja et al. 2014). Water table can be affected by human-induced hydrological changes occurred on the peatland’s catchment, therefore disturbing or changing the function of an original peatland ecosystem (Tahvanainen 2011). Due to peatlands’ and other wetlands’ dependence on water, their protection can be effective in a long term only when their hydrological processes are safeguarded which often means setting aside relatively large continuous areas. On many wetland types, land-use pressures are or have historically been high so often they are degraded and/or exposed to an intensive competition between different forms of land use (e.g.,Vasander et al. 2003; Pin et al. 2011). Furthermore, in several countries such as the UK, the USA, and Finland, most of the wetlands are privately owned (Brown et al. 2001; Bain et al. 2011; Alanen and Aapala 2015). For these reasons, protecting large continuous areas can be challenging in terms of economy and/or social acceptability.

Voluntary conservation approaches (Kamal et al. 2015) have positive effects in biodiversity protection both in an ecological and a social manner: they have e.g., decreased forest loss and development (Nolte et al. 2019), targeted priority areas of biodiversity (Fisher and Dills 2012), and tackled biodiversity conflicts between private landowners and environmental governance (Paloniemi and Tikka 2008). The downside of focusing conservation efforts only on lands of conservation-minded owners seems to be the trade-offs that exist between landowners’ preferences and conservation targets, causing the latter not to be efficiently met (Guerrero et al. 2010; Knight et al. 2011; Adams et al. 2014). Landowners can be persuaded to participate in conservation initiatives by paying more (e.g., Sorice et al. 2013), which increases conservation costs without still necessarily achieving the same biodiversity level to be protected than if an optimal set of areas could be freely chosen for protection (Lewis et al. 2011).

Systematic spatial prioritization is a land-use planning approach where divergent land-use needs and restrictions can be attempted to match together and potential trade-offs between them to be quantified (e.g., Moilanen et al. 2011). In a spatial conservation prioritization, utilization of biodiversity information has traditionally received a major role (Knight and Cowling 2007). In a real-world conservation decision-making, however, it is not the ecological considerations alone, but the social, financial, political, and ecological factors together that determine the final conservation implementation (Kareksela et al. 2018). Hence, inclusion of societal aspects in conservation prioritization is needed to promote the uptake of the results (Ban et al. 2013). Integration of economic costs has been customary for over a decade (Naidoo et al. 2006), but only during the recent years other societal aspects such as alternative political scenarios (Di Minin et al. 2017), values and preferences of people (Whitehead et al. 2014; Brown et al. 2019), and private landowners’ willingness to participate (Guerrero et al. 2010; Knight et al. 2011; Adams et al. 2014; Nielsen et al. 2016; Paloniemi et al. 2018) have been incorporated into spatial conservation prioritization.

When protecting private land, considering landowners’ perspectives to protection is frequent, but its effects on conservation outcomes are not often quantitatively reported (but see Guerrero et al. 2010; Knight et al. 2011; Adams et al. 2014). To our knowledge, potential trade-offs related to a level of protected biodiversity, financial costs of conservation, and citizen landowners’ conservation preferences have not been previously studied in the context of peatlands (wetland ecosystems, e.g., mires, that actively form peat). In this paper, we detected and quantified potential trade-offs arising from voluntariness requirement of protection by comparing three alternative spatial prioritizations in the case of complementing the network for protected mires in Finland. Our work was motivated by the challenges faced in the implementation of the Complementary Mire Protection Program (hereafter CMPP; Alanen and Aapala 2015; Kareksela et al. 2020). Originally, CMPP was based on the Nature Conservation Act allowing land expropriations for conservation purposes if a landowner would have resisted establishing protected area on his/her land. The Act also obligates the government to pay a full economic compensation to landowners, whether their lands are protected based on a voluntariness or expropriations. The spatial prioritization aiming to select mires for CMPP was made like all the mires chosen as candidates for protection were available (Kareksela et al. 2020), without paying attention to landowners’ preferences. Just before CMPP implementation was about to be started, the government changed its politics and revised CMPP to be based purely on landowners’ willingness to protect (the compensation practice staying equal), which changed the preparation and the prioritization problem of CMPP remarkably.

We integrated data of landowners’ resistance to their lands’ protection into spatial prioritization of CMPP site selection. We first compared the results of two prioritization analyses. In the first one, expropriations of mire properties owned by conservation-resistant owners were allowed. In the second one, only mires owned completely by conservation-minded landowners could be protected. This excluded from protection such mires that were owned both by willing and unwilling landowners, representing the need to set aside mires as hydrological entities to achieve long-term effectiveness in their protection. These two prioritizations imitated the situations before and after CMPP was revised to be a voluntary program. Next, we designed a prioritization model that allowed, but aimed to minimize, the protection of resisting landowners’ land, while cost-efficiently trying to maximize the level of biodiversity representation in the prospective mire conservation network. Comparisons between all the three prioritizations allowed us to quantify how the trade-offs concerning the average protected representation of biodiversity features, conservation costs, and landowners’ resistance to protection varied depending on how much the resistance was allowed to limit the prioritization. Our analysis shows that although trade-offs between ecological, economic, and social matters faced by biodiversity protection cannot be fully avoided, there are still opportunities to alleviate them. Our prioritization analysis and discussion drawn from its results can be applicable for other large-scale conservation initiatives that aim to protect mostly privately owned wetlands, peatlands, or other such ecosystems that often require setting aside large continuous areas in order to maintain their functions.

## Materials and methods

### Study case

This study considers mire ecosystems as a case. ‘Mire’ is a general term for natural and semi-natural peatlands actively forming peat (Lindsay 2018). When preparing CMPP in Finland, approximately 117 000 ha of unprotected mires were to be assigned for protection (Alanen and Aapala 2015). The spatial prioritization aiming to select mires for protection covered totally 929 000 ha, including the candidate mires for protection (327 300 ha) and the already protected mires (601 700 ha) (Alanen and Aapala 2015). Including the existing mire conservation network into prioritizations enabled to detect biodiversity features poorly covered by the current network and to select for protection such mires that would potentially retain them (Kareksela et al. 2020).

Originally, there was a political agreement to implement CMPP according to the Nature Conservation Act, which allows land expropriations for conservation purposes without landowners’ consent (Salomaa et al. 2018). Regardless of whether private land is protected voluntarily or via expropriations, the Act requires all protected sites to be economically fully compensated to landowners from public funds (Alanen and Aapala 2015). Additionally, public rights of access are very broad in Finland ensuring that landowners and all other citizens are allowed e.g., to visit private sites and pick berries and mushrooms there, regardless of whether the sites are protected or not (Ministry of the Environment 2016). Due to political changes, however, the possibility of land expropriations was rejected from CMPP (Salomaa et al. 2018). In the new situation, landowners’ consent was needed for protection of their land. In public deliberation, the rejection was praised for acknowledging private property rights, but also criticized because voluntarily protecting mires as hydrological entities was seen potentially challenging as landownership in Finland is very fragmented and majority of the candidate mires are owned by more than one person.

### Data

Biodiversity data contained 91 feature layers including mire complexes, i.e., geomorphological form of a mire pool (31 layers), mire habitat types (39), small waters (1), plants and mosses (1 layer for critically endangered, endangered and vulnerable species, 1 for near threatened, regionally threatened and data deficient species, and 1 for least concerned but otherwise interesting species), and modeled likelihoods of occurrences of birds favoring mire habitats (17). Cost data layer (1) was based on each mire’s financial compensation value, including the value of land area, tree stand, and an administrative cost (Alanen and Aapala 2015). Mire-specific ditching level, i.e., proportion of area ditched, served as a habitat condition layer (1) reflecting the ecological intactness lowered by drainage pressure. See Kareksela et al. (2020) for a detailed description of the biodiversity and condition data.

Data on landowners’ resistance to protection (1 layer) were based on a landowner survey and on negotiations with forestry and peat mining companies, both implemented by Finnish Ministry of the Environment. Survey was sent to the citizen owners of 562 candidate mires that were proposed for protection. One of the questions asked was: “Would you consider protecting your property shown in an attached map for the market price?” In the time of performing the survey, prices of sites were not yet calculated. Answer options were “Yes”, “I don’t know”, and “No”. We considered all “No” answers as a resistance to protection and all “Yes” and “I don’t know” answers as a positive attitude. Due to the lack of time, Ministry of the Environment decided the survey would be sent only to citizen owners of those candidate mires that were suggested for protection and located in the 10 most southern provinces (Alanen and Aapala 2015; Appendix S1, Fig. S1; Table S1). In addition, 58% of survey recipients did not answer. Therefore, using only the observed preferences of landowners would have restricted the analysis area impractical small for prioritizations so to get a full data coverage, we extrapolated citizen landowners’ observed resistance to protection to cover all the candidate mires owned by citizens. As landowners’ attitude toward conservation can depend on their relationship to regional environmental authorities (Salomaa et al. 2016), we made extrapolations independently for each of the 10 southern provinces, based on their observed distributions of resistance. In each province, we calculated the resistance distribution from the observed mire-specific resistances. Following the distributions, we then randomized the resistance percentages to the mires lacking observed resistance. Three northern provinces were totally excluded from the survey, so for candidate mires located on them, we randomized the percentual average resistances following the combined observed resistance distribution of all 10 southern provinces included to the survey. Finally, we had a resistance layer including both the observed resistance of citizen-, state-, and company-owned candidate mires and the extrapolated resistance of those citizen-owned candidate mires, which were not covered by the observed resistance data. For more information of the landowner data and extrapolation, see Appendix S1.

We investigated correlations between the extrapolated resistance data and biodiversity, cost, and area data to see, if the extrapolation process created any random correlations between the datasets. No correlations were found (see Appendix S2).

All data layers were converted to 50 × 50 m raster data.

### Spatial prioritizations

**Fig. 1:**
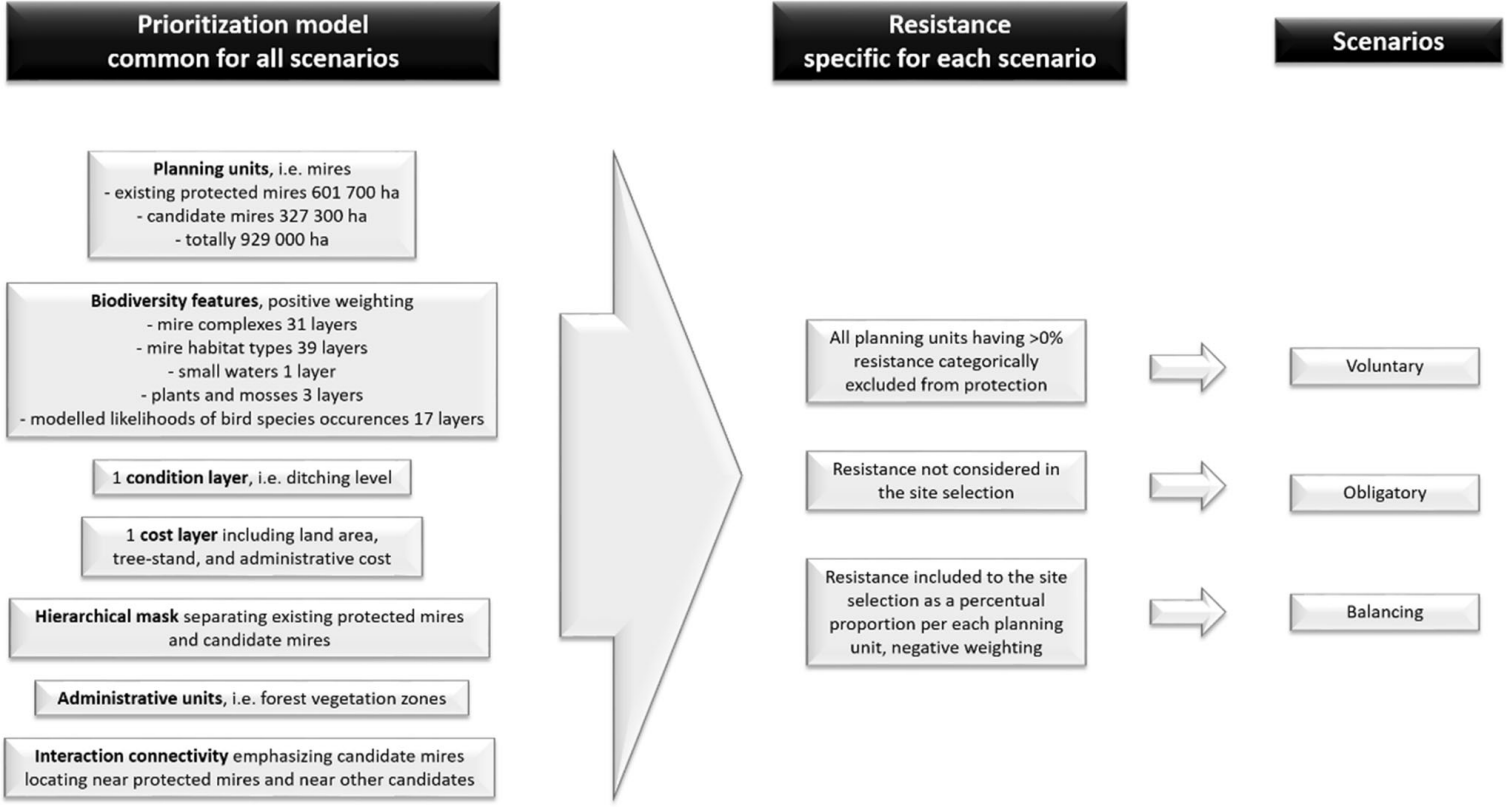
Schematic visualization of the prioritizations of the three scenarios (*Voluntary*, *Obligatory*, and *Balancing* ones). For a detailed description, see text and Kareksela et al. (2020)

Spatial prioritizations were made using Zonation software 4.0.0 (Moilanen et al. 2005, 2014). Mires were prioritized as planning units forming hydrologically connected entities, defined by mire experts. Prioritization model also included weights for the biodiversity features (see Appendix S3); a hierarchical mask to separate candidate mires from the existing protected mire network and enabling emphasis of those candidate mires whose features best complement the already protected mires (Appendix S3); administrative units, i.e., forest vegetation zones, to consider both local and national scale rarity of the biodiversity features (Appendix S3); and an interaction connectivity to emphasize the candidate mires that locate near already protected mires or near other candidate mires, aiming to ensure ecological connectivity (Fig. 1). Additionally, a cost layer and a condition layer were applied to de-emphasize high-priced mires and mires with lowered ecological condition due to ditches, respectively. For a more detailed description of the prioritization model, see Kareksela et al. (2020).

For three different prioritizations, we designed three scenarios that differed in how they considered landowners’ resistance to protection. In *Voluntary scenario*, all candidate mires having at least one resisting owner (either a citizen or a company) were excluded from protection regardless of their average biodiversity feature representation or financial costs. In *Obligatory scenario*, mires with high feature representation but low costs were aimed, ignoring landowners’ resistance to protection. In *Balancing scenario*, resistance was considered as a continuous variable, i.e., proportion of each candidate mire’s landowners resisting protection. Here, we gave a negative weight to resistance in order to de-emphasize resisted mires in prioritization. To determine a suitable level of negative weight for resistance, we iterated the analysis with several differing levels of weighting and investigated the related trade-offs (Appendix S3). For this example case, we were able to find a value resulting in a solution with relatively small loss in biodiversity representation, but significantly lower landowners’ resistance than *Obligatory scenario*. In other words, *Balancing scenario* aimed simultaneously to avoid resisted mires while still emphasizing high overall feature representation and low costs.

### Comparison between scenarios

We compared the scenarios by observing their levels of average biodiversity feature representation protected (% of total feature representation included in prioritizations), costs (million euros), and resistance (% of all landowners). To make comparisons comprehensible, we chose *Voluntary scenario* as a reference for two other scenarios because it explicitly defines the candidate mires that are completely free from landowners’ resistance to protection. We compared *Obligatory* and *Balancing scenarios*’ average feature representation, costs, and total protected area against those set by *Voluntary scenario*.

## Results

**Fig. 2:**
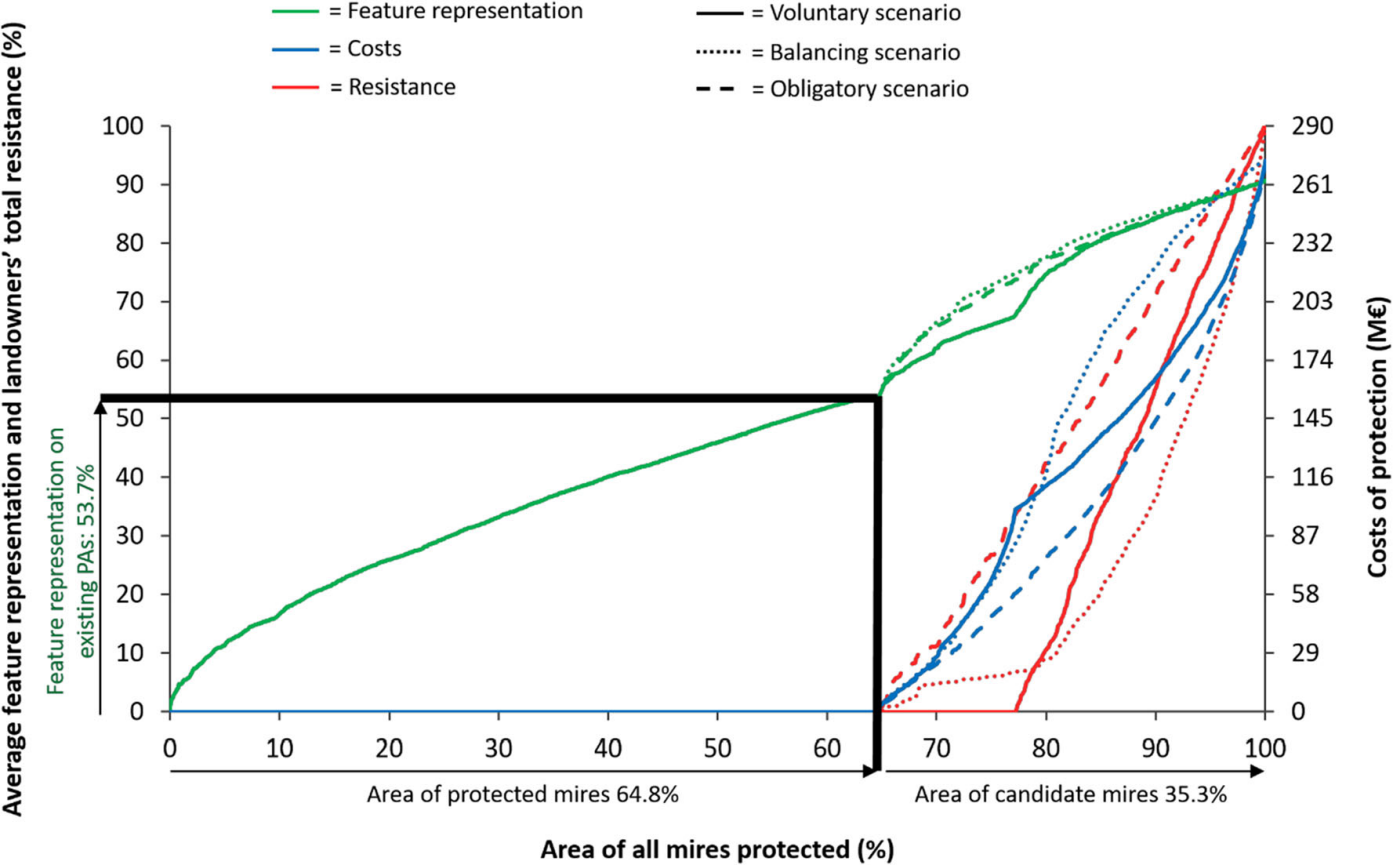
Proportion of the existing protected mire network and the candidate mires and the biodiversity feature representation covered by the existing network. Thick black lines represent the average representation of biodiversity features (53.7%) and total area (64.8%) covered by existing protected mire network. Green curves represent average feature representation, red curves landowners’ total resistance to protection, and blue curves conservation costs in each scenario. Solid colored lines represent *Voluntary scenario*, colored dotted lines *Balancing scenario*, and colored dashed lines *Obligatory scenario*. Average feature representation does not reach 100% because the included mire complexes and habitats suffer from decreased condition caused by drainage, expressed by the curves

Of the total 929 000 ha included in the prioritizations, the existing protected mire network covered 64.8% (601 700 ha) and the candidate mires 35.2% (327 300 ha) (Fig. 2). 53.7% of average representation of biodiversity features included in the prioritizations were situated on the existing protected mire network (Fig. 2; Table 1).

**Table 1:** Average representation of biodiversity features, financial costs, and area of existing protected mire network and *Voluntary*, *Balancing,* and *Obligatory scenarios* when average feature representation, costs, or area has been fixed according to the values produced by *Voluntary scenario*

	Average feature representation fixed				Costs fixed				Area fixed			
	Biodiversity %^a^	Cost M€^b^	Area % (ha)^a^	Resistance %^b^	Biodiversity %^a^	Cost M€^b^	Area % (ha)^a^	Resistance %^b^	Biodiversity %^a^	Cost M€^b^	Area % (ha)^a^	Resistance %^b^
Existing PAs	53.7 | na	na	64.8 | na (601 700 | na)	na	53.7 | na	na	64.8 | na (601 700 | na)	na	53.7 | na	na	64.8 | na (601 700 | na)	na
Voluntary scenario	67.7 | 37.2	97.9	77.2 | 35.3 (717 100 | 115 400)	0	67.7 | 37.2	97.9	77.2 | 35.3 (717 100 | 115 400)	0	67.7 | 37.2	97.9	77.2 | 35.3 (717 100 | 115 400)	0
Balancing scenario	67.8 | 37.3^c^	31.5	71.2 | 18.3 (661 500 | 59 800)	5.0	76.0 | 60.1	97.8^c^	78.5 | 39.1 (729 200 | 127 500)	7.2	74.8 | 56.7	80.8	77.2 | 35.3 (717 100 | 115 400)	6.7
Obligatory scenario	67.7 | 37.2	27.8	71.5 | 19.1 (664 200 | 62 500)	16.8	80.0 | 70.8	97.8^c^	84.0 | 54.6 (780 300 | 178 600)	52.5	73.8 | 53.7	55.8	77.1^c^ | 35.1 (716 400 | 114 700)^c^	33.7

^a^The first number represents %-values (hectares in brackets) after protection (existing PAs + selected candidate mires), while the second number after the vertical line shows the %-values (hectares in brackets) of the candidate mires that were included into the scenarios

^b^The numbers represent values calculated for the candidate mires that were included into the scenarios

^c^As the prioritization was conducted in planning units, the area or costs cannot be fixed exactly the same in each scenario (i.e., cut in the middle of a planning unit). This results small variation to the fixed numbers

*Voluntary scenario*, allocating to protection all candidate mires completely free from landowners’ resistance, increased the average protected representation of biodiversity features by 26.1% (from present 53.7 to 67.7%), with the costs of 97.9 million euros (Table 1, Appendix S4, Fig. S3). Protected total area increased with 115 400 ha (19.2%), which is close to the original proposal (117 000 ha) (Alanen and Aapala 2015).

With the same average representation of biodiversity feature as in *Voluntary scenario*, the area protected and the costs needed to protect it were much lower both in *Balancing* and *Obligatory scenarios* than in *Voluntary scenario*. Both *Balancing* and *Obligatory scenarios* were able to fulfill the average feature representation of *Voluntary scenario* by almost half the area (59 800 and 62 500 ha vs. 115 400, respectively) (Table 1, Appendix S4, Fig. S3a). Accordingly, the costs of protection decreased remarkably. *Balancing scenario* paid 66.4 million euros less and *Obligatory scenario* 70.1 million euros less than *Voluntary scenario*. *Balancing scenario* performed well in keeping the total landowners’ resistance low at 5.0%, while in *Obligatory scenario* the total resistance was 16.8%.

With the same budget for protection as in *Voluntary scenario*, *Balancing scenario* resulted in 10.5% larger total area for protection with 12.3% higher average feature representation than *Voluntary scenario* (Table 1, Appendix S4, Fig. S3b). Total resistance still stayed relatively low at 7.2%. In *Obligatory scenario*, total area protected was 54.8% larger and the average feature representation 18.2% higher than in *Voluntary scenario*. Area protected covered already over half of the candidate mires (176 600 ha) and the total resistance increased to 52.5%.

With the same total area protected as in *Voluntary scenario* (115 400 ha), *Balancing scenario* achieved 10.5% higher average feature representation with 17.1 million euros lower costs than *Voluntary scenario* (Table 1, Appendix S4, Fig. S3c). *Balancing scenario* performed rather well also in minimizing the total resistance as it remained relatively low at 6.7%. Relative to *Voluntary scenario*, *Obligatory scenario* covered 9.0% more average feature representation with 42.1 million euros lower costs. However, the total resistance increased to 33.7%.

## Discussion

In this paper, we have demonstrated that when selecting sites for protection, it is possible not only to consider different social, ecological, and economic aspects, but also to identify and quantify trade-offs between them. This allows providing detailed information of the related compromises for the decision-making process (see also e.g.,Guerrero et al. 2010; Knight et al. 2011; Adams et al. 2014; Kareksela et al. 2020).

In our case of boreal mire protection, the dimensions of the trade-offs between the level of average protected biodiversity features, financial costs of conservation, and the amount of landowners’ resistance to protection varied remarkably depending on how the landowners’ resistance was considered in the prioritization. *Balancing* and *Obligatory scenarios* were able to protect the same level of average feature representation than *Voluntary scenario* with lower costs and smaller area. When their total cost was restricted to that of *Voluntary scenario*, they protected more biodiversity and more area. When total area protected in them was restricted to the one available for protection in *Voluntary scenario*, they protected more biodiversity with lower costs. In summary, cost-efficiency was remarkably low in *Voluntary scenario*. This is problematic as conservation resources are often scarce with respect to conservation aims and requirements (Geldmann et al. 2018), meaning that less money would be available for other conservation purposes and still, the biodiversity effect of the scenario is inferior compared to the other scenarios (see also Lewis et al. 2011).

Importantly, however, *Balancing scenario* aiming to simultaneously both minimize landowners’ resistance to protection and cost-efficiently maximize representation of biodiversity features seemed to reach its goals. It was able to achieve nearly the same average feature representation as *Obligatory scenario*, while it allocated significantly less lands of conservation-resistant landowners to protection. Its costs were intermediate compared to other scenarios meaning that its cost-efficiency was higher than that of *Voluntary scenario*.

Regardless of *Balancing scenario*’*s* ability to alleviate the trade-offs, it should be noted that it is worthy of its name mainly from a wider societal perspective. For single resisting landowners, enforcing protection still represents an extreme. Land expropriations are made for a compelling public need such as large infrastructure initiatives, and they typically cause a conflict between individuals’ rights and the needs of the society. In the case of biodiversity conservation, expropriations may also cause intentional harming of biodiversity features by landowners if they try to avoid protection (Lueck and Michael 2003; Simmons et al. 2018). In Finland, expropriations for conservation purposes have been less restrictive from landowners’ point of view than expropriations for the most infrastructure initiatives as people have not been displaced in consequence of conservation. Additionally, broad public rights of access ensure an entrance to private land with certain constraints regardless of whether the land is protected or not, which could alleviate the experienced harm caused by enforced protection (Ministry of the Environment 2016).

Since different forms of voluntary conservation approaches facilitate acceptance of conservation amongst local people and make protection more successful in the long run (e.g., Paloniemi and Tikka 2008), also lands of the remaining resisting landowners in *Balancing scenario* should preferably be protected via voluntary means. Conservation willingness can be boosted e.g., by improving landowners’ knowledge and convincing them of the high ecological value of their land since they may not know or understand the lack of compensatory sites concerning endangered biodiversity features (Olive and McCune 2017). The method used in this study can serve as a tool to make the trade-offs concrete to stakeholders by explicitly visualizing them (see also e.g., Kareksela et al. 2020). Additionally, further increasing the level of financial compensation could persuade unwilling landowners to become more receptive to conservation (e.g., Sorice et al. 2013). In our case, the financial compensation for landowners was a uniform payment based on an area of a mire and, if relevant, on an economic value of its tree stand. Instead of a uniform payment, designing an agglomeration bonus mechanism could persuade landowners to protect a well-connected habitat ensemble (Parkhurst et al. 2002; Drechsler et al. 2010; for further discussion, see also Nieminen 2020). Such incentive mechanisms could alleviate the challenge that fragmented landownership causes in protecting habitats requiring spatial connectedness and therefore, could provide higher ecological profit compared to a uniform payment. It needs to be noted, however, that even a large compensation may not satisfy all landowners since other than economic reasons such as place attachment (Selinske et al. 2015) or dislike of government regulation (Olive and McCune 2017) cannot be compensated with money. Still in our case, cost differences between *Voluntary* and two other scenarios raise an interesting option to increase resistant landowners’ conservation willingness by paying more. In *Obligatory* and *Balancing scenarios*, landowners of the most biodiversity-rich mires resisting protection could be persuaded to protect by increasing the compensation level multifold from their current level before their total costs would match those of *Voluntary scenario*.

Overall, successfully implementing large-scale protection of mires or other structurally connected ecosystems should follow several steps that are included e.g., in the Systematic Conservation Planning framework (Margules and Pressey 2000). First, landowners’ preferences should be acknowledged in a very early planning stage, thus enabling ecologically effective site selection while aiming at avoiding resisted sites. Second, trade-offs between different aspects and their possible solutions should be thoroughly analyzed and quantified. Third, enough resources should be allocated to ensure protection of the sites that host irreplaceable biodiversity features or have otherwise a significantly high conservation effect. Consequently, it could be possible to reduce the likelihood of the somewhat undemocratic situation seen in *Voluntary scenario*, where a single unwilling landowner among many willing ones shifts a whole mire to be out of reach for protection, causing conservation opportunities to be lost or diminished for the society. Obviously, reconciling multiple differing needs of various groups of people and multiple requirements of various biodiversity features will never be free from practical and moral challenges (Batavia et al. 2020), but as we have shown, many of the challenges can be alleviated and resolved.

## Conclusions

Peatlands and other wetlands should be protected as hydrologically connected and functional entities, which makes their conservation challenging especially if they are largely privately owned and/or their landownership is fragmented. When making a systematic reserve site prioritization for such ecosystems, categorical exclusion of sites owned by even one person that resist biodiversity conservation on her/his land leads to an ecologically and economically inefficient conservation solution, whereas ignoring landowners’ preferences can fortify conservation conflicts by including multitude of resistant landowners’ lands into the conservation solution. Instead, a recommended approach would be to make a reserve site selection with a prioritization model that aims to minimize inclusion of resistant landowners’ lands while simultaneously cost-efficiently maximizing the representation of protected biodiversity features. The model allows determining the conservation solution that is based on sites owned by conservation-minded landowners, while recognizing the sites that are particularly important or irreplaceable for biodiversity, but face resistance to protection by landowners. If the most important sites can be protected e.g., via allocating more conservation resources on them, the conservation solution reaches higher cost-efficiency than the solution categorically excluding all the resisted sites, but still has high ecological gains as the solution ignoring landowners’ preferences. At large, quantification of the trade-offs arising from biodiversity protection helps to predict and evaluate conservation policy outcomes, enabling better-informed conservation decisions. Tools such as the demonstrated prioritization model that alleviates trade-offs between different aspects has a potential to assist practical conservation planning and enable ecologically, socially, and economically effective biodiversity conservation.

## Electronic supplementary material

Below is the link to the electronic supplementary material.

(PDF 807 kb)
