# Supplementary material for: Quantifying trade-offs between ecological gains, economic costs, and landowners’ preferences in boreal mire protection
Source: Ambio. 2021 Apr 6;50(10):1841–50. doi: 10.1007/s13280-021-01530-0 (PMC8363685; doi:10.1007/s13280-021-01530-0)
Supplement: Supplementary file 1 — (PDF 807 kb) [file 13280_2021_1530_MOESM1_ESM.pdf]

Ambio

Electronic Supplementary Material
This supplementary material has not been peer reviewed.

Title: Quantifying trade-offs between ecological gains, economic costs, and landowners' preferences in boreal mire protection

Authors: Eini Nieminen, Santtu Kareksela, Panu Halme, Janne S. Kotiaho

Appendix S1

Survey data

Survey concerning citizen landowners' perceptions of protecting their estates was assigned by Ministry of the Environment after the option for land expropriations was rejected. 10 of 13 provincial Centres for Economic Development, Transport and the Environment (ELY Centres) implemented the survey in spring 2015. It was sent only to the citizen landowners of those candidate mires that were finally planned to be protected (*n*=562, Table S1 and Fig. S1) (Alanen & Aapala 2015), excluding the candidate mires planned to be left outside protection. Therefore, on the territories of 10 ELY Centres about half of the mires (*n*=508) were excluded from the survey. Three northernmost ELY Centres did not implement the survey at all (*n*=384) because of the lack of time and because perceptions of landowners of the southern candidate mires were thought to be the most urgent to ask due to the alarming state of mires' biodiversity in Southern Finland and their high proportion of private land. 42% of the landowners answered the survey: positive and negative attitudes towards conservation were 47% and 41%, respectively, and 12% were not sure about their opinion (Alanen & Aapala 2015). Protection of sites owned by forestry or peat mining companies or the state of Finland was negotiated between them and the Ministry of the Environment, and the mire-specific results of the negotiations were included to the observed landowners' resistance to protection.

ELY Centres had slightly different practices in implementing the survey and recording answers. Some sent the survey letter to only one landowner per each estate, while others send the letters to all landowners. Some ELY Centres registered answers of a single estate, if at least one of an estate's owner responded, while others registered answers only when all landowners of an estate had the same perception towards conservation. Due to incoherence, we needed to simplify the observed survey data. We assumed that the number of sent survey letters corresponded to the number of estates per single candidate mire and that each estate had one voice for or against protection.

Hence, the assumed number of estates is probably larger than the real number of estates (Table S2). In the processed survey data, positive and negative perceptions were 47.7 and 41.2%, respectively (Table S3).

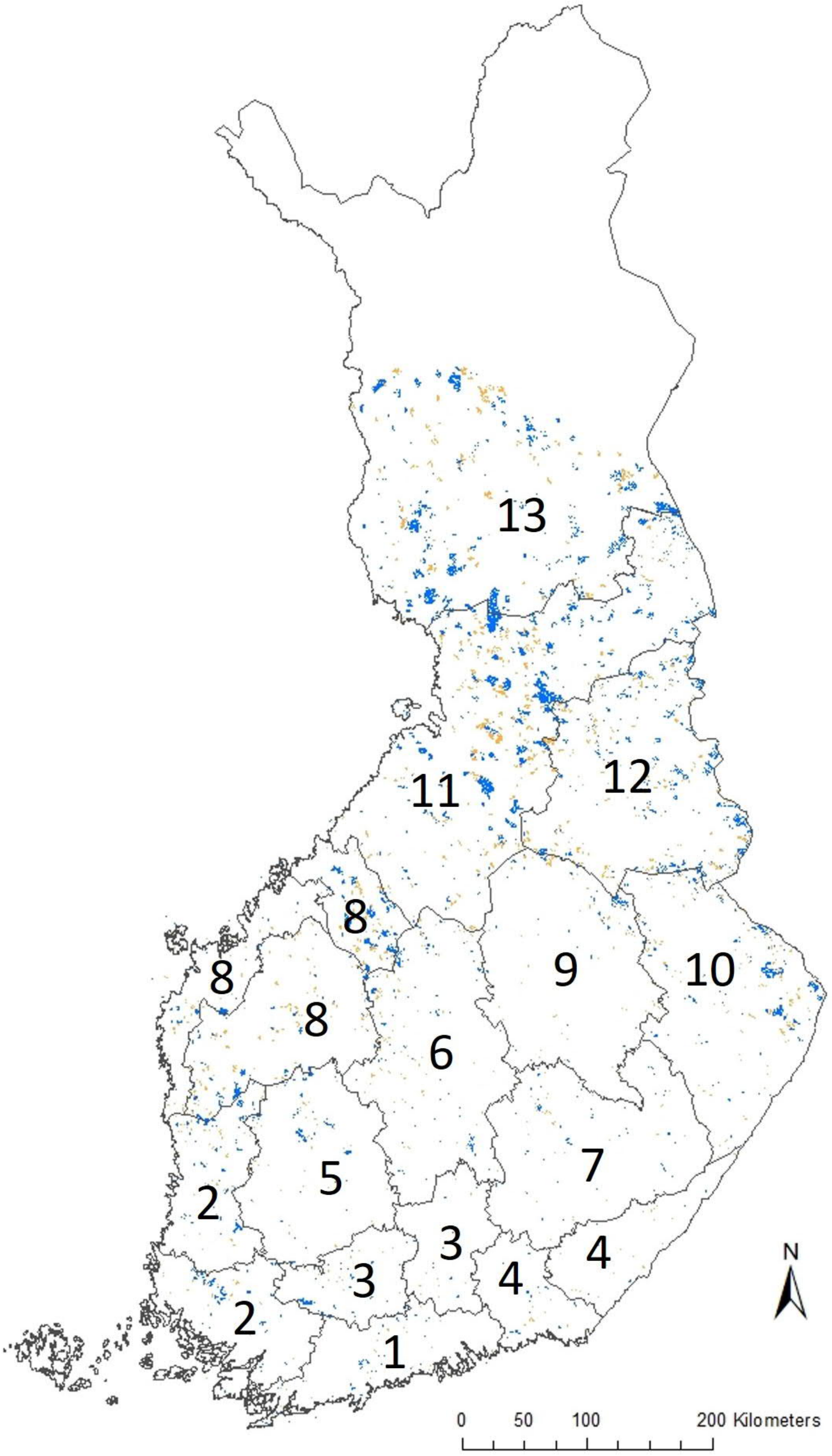

Figure S1. Existing protected mires (blue), candidate mires (yellow), borders of provinces, and numbers indicating regional ELY Centres (see also Table S1). Some of the ELY Centres implemented the landowner survey in several provinces. The Complementary Mire Protection Program (CMPP) did not cover the northern Lapland and Åland Islands in south-west.

Table S1. Province-specific information of landowners' preferences concerning protection of their land in the processed survey data.

*Province	Mires included (of which no answers)	Mires excluded	Companies or state owned mires		Observed avg resistance %	Extrapolated avg resistance %
1. Uusimaa	42 (6)	18	0		25.1	19.0
2. South-West Finland	75 (7)	58	0		17.9	16.9
3. Tavastland	45 (3)	36	5		18.2	20.0
4. South-East Finland	52 (7)	33	3		20.1	20.9
5. Pirkanmaa	57 (3)	24	4		20.5	23.7
6. Central Finland	59 (11)	92	9		14.3	11.7
7. South Savo	54 (6)	19	3		12.1	17.6
8. South Ostrobothnia	66 (1)	125	1		29.9	30.6
9. North Savo	56 (2)	27	5		16.6	14.3
10. North Karelia	56 (11)	76	11		12.4	12.2
11. North Ostrobothnia	0 (0)	180	0			
12. Kainuu	0 (0)	138	34		NA	18.4
13. Lapland	0 (0)	66	4			
Total	562 (57)	892	79	Average	18.7	18.7

* Numbers and names of the provinces corresponds with the map in Fig. S1.

Table S2. Distribution of area and estates between different forms of landowning in the processed survey data.

	Citizen landowners		Private companies		State of Finland		Total	
	Number	%	Number	%	Number	%	Number	%
Hectares	36 511	24.9	12 088	8.2	98 083	66.9	146 682	100.0
Estates	5 436	88.5	193	3.1	516	8.4	6 145	100.0

Table S3. Number of landowners, number of total answers received from all of the landowners, and number and percent of positive, negative, and unsure perceptions towards conservation in each group of different landowning form.

	Landowners	Total answers		Positive answers		Negative answers		Unsure answers	
	n	n	%	n	%	n	%	n	%
Citizen landowners	5 436	2 260	100.0	1 078	47.7	932	41.2	250	11.1
Private companies	3	193	100.0	183	94.8	10	5.2	0	0.0
The state	1	516	100.0	516	100.0	0	0.0	0	0.0

Extrapolation of the survey data

Altogether 892 candidate mires were excluded from the survey and the data was deficient for 57 included candidate mires due to lacking survey answers (Table S1). Due to the shortages, we extrapolated citizen respondents' resistance to protection to cover mires lacking the observed data.

Whether citizen landowners would voluntarily protect their land or resist protection may depend on their relationship to regional advisors and authors (Salomaa et al. 2016). Presumably, landowners living in the same region have more parallel attitudes towards conservation than randomly picked landowners do. Provinces included to the survey had different levels of resistance (Table S1). Therefore, for the mires of such provinces where at least part of the mires were included to the survey (provinces 1–10 in Fig. S1 and Table S1), we extrapolated citizen landowners' resistance to protection based on the observed survey data. We calculated average resistance for each mire of which we had observed resistance data. For the mires lacking the observed data, we extrapolated the resistance randomly from the resistance distribution of the mires within the province in question. For the provinces that were totally excluded from the survey (provinces 11–13 in Fig. S1 and Table S1), we repeated the same procedure, but used the observed average resistance distribution of all the provinces included to the survey. We did randomization by Shuffle tool of Microsoft Excel 2016.

References

Alanen, A., and Aapala, K. (Eds.). 2015. Proposal of the Mire Conservation Group for supplemental mire conservation. Reports of the Ministry of the Environment 26 | 2015. http://hdl.handle.net/10138/158285 (in Finnish, English summary).

Salomaa, A., Paloniemi, R., Hujala, T., Rantala, S., Arponen, A., and Niemelä, J. 2016. The use of knowledge in evidence-informed voluntary conservation of Finnish forests. *Forest Policy and Economics* 73: 90–98. https://doi.org/10.1016/j.forpol.2016.09.004

Appendix S2

Correlations in the data

For background knowledge, we tested correlations of the candidate mires' area, price, resistance, and conservation priority using two-way Pearson correlation (Table S1). While preparing the analysis in phases, with respect to the used data and other considerations (see e.g. Kareksela et al. 2013), we also constructed an analysis version with all the presented biodiversity features included, but without connectivity, costs, and resistance considered. We used the rank order of the candidate mires of this analysis version as a variable for conservation priority.

Area and cost of the candidate mires correlated negatively likely because large mires are commonly treeless and therefore their acquire costs are lower than those of small mires (Table S1). Conservation priority and costs of the candidate mires correlated positively. This is intuitive as more fertile mires have higher tree growth, higher species richness, and they consist of more threatened habitat types. Area and conservation priority of the candidate mires correlated negatively, likely because large mires always include both highly and less valuable biodiversity features, so the average conservation value in relation to area is lower on large than on small mires. Landowners' resistance to protection did not correlate with any variable.

Table S1. Correlations between the candidate mires' area, cost, resistance and conservation priority.

Correlation pairs	n	Coefficient	95% CI	*p*-value
Area vs. resistance	437	0.049	−0.04, 0.14	0.304
Area vs. cost	1 533	−0.266	−0.31, −0.21	0.000
Cost vs. resistance	437	−0.045	−0.14, 0.05	0.347
Biodiversity vs. resistance	437	−0.020	−0.11, 0.07	0.683
Biodiversity vs. cost	1 533	0.270	0.22, 0.32	0.000
Biodiversity vs. area	1 533	−0.237	−0.28, −0.19	0.000

References

Kareksela, S., Moilanen, A., Tuominen, S., and Kotiaho, J.S. 2013. Use of Inverse Spatial Conservation Prioritization to Avoid Biological Diversity Loss Outside Protected Areas. *Conservation Biology* 27: 1294–1303. https://doi.org/10.1111/cobi.12146

Appendix S3

Weighing of features on local and national scale (from Kareksela et al. 2020)

Weights for the features inside feature groups (e.g. mire complexes, mire habitats, threatened mosses and vascular plants, birds, streams and bonds) were following the features' Red List status classifications (Raunio et al. 2008, Rassi et al. 2010) and the between biodiversity feature group importance was defined together with the experts in the working group (Tables S1 and S2). For the features lacking Red List status, the weight was based on their relative importance according to the experts' opinion. The expert working group comprised 14 stakeholders and experts, including experts of mire ecology, land-use planners, conservation scientists, and representatives of the environmental and forestry administration, the land owner's association and conservation NGOs.

Table S1. Biodiversity features included in the analysis and their national conservation status, national weights (defined by the experts for the analysis), number of cells and proportion remaining before prioritization after corrected by condition (the ditching level). Condition was applied only to habitat complexes and types. Some of the names for the mire complexes do not have English equivalents.

Mire habitat complexes	Status	Weight	Number of cells	Proportion remaining before prioritization
northern boreal aapa mires	LC	3	485 523	0.9638317
northern boreal sloping fens	LC	3	41 287	0.9474729
Transition states between the mire complex forms	-	3	150 134	0.8004229
Not identified	-	3	39 468	0.4814771
concentric raised bogs	NT	6	235 750	0.8361712
plateau raised bogs	NT	6	1 200	0.7496354
eccentric raised bogs	VU	9	150 790	0.8783805
Sphagnum fuscum raised bogs	VU	9	263 120	0.8293616
middle boreal flark-surfaced aapa mires	VU	9	764 146	0.9101144
middle boreal sloping fens	VU	9	15 000	0.8654042
seasonal wetlands	DD	9	194	0.8811211
harjunlievesuot	-	9	1 028	0.8722033
suppasuot	-	9	1 863	0.9556830
kumpumoreenialueisiin liittyvät suot	-	9	2 058	0.9664723
jokimeanderisuot	-	9	427	0.9705796
virtavesien rantasuot	-	9	32 799	0.7522600
lampien ja järvien rantasuot	-	9	22 584	0.8680648
jokiterassisuot, joki- ja purolaaksojen suot	-	9	7 033	0.6612576
rannikon ja saariston nuoret suot ja soiden kehityssarjat	-	9	2 201	0.9492276
puustoiset piensuot, erityisesti korvet	-	9	222 629	0.8310140

letot	-	9	13 285	0.8863333
lähteiköt, lähdesuot ja muut pohjavesivaikutteiset suot	-	9	1 694	0.7543536
paikalliset suoyhdistymät	-	9	477 941	0.8736399
suometsämosaiikit	-	9	6 072	0.8948555
kalliopainannesuot	-	9	9 506	0.9499724
rantavalli- ja dyynikenttien suot	-	9	7 409	0.8634009
vesistönlaskusuot	-	9	15 958	0.8132011
pienet avosuot	-	9	22 773	0.8774041
wooded raised bogs	EN	12	3 230	0.8445434
middle boreal lawn-surfaced aapa mires	EN	12	817 119	0.8565504
mire succession series of the land uplift coast	CR	15	27 406	0.9372355
Mire habitats				
hollow bogs	LC	3	1 114 925	0.8532036
flark fens	LC	3	1 781 350	0.8228307
swamp fens	LC	3	1 331 718	0.8860366
ombrotrophic low-sedge bogs	LC	3	1 523 134	0.8426581
minerotrophic low-sedge fens	LC	3	2 449 233	0.8588128
tall-sedge fens	LC	3	2 711 144	0.8597798
Sphagnum fuscum bogs	LC	3	2 561 744	0.8520335
dwarf shrub pine bogs	LC	3	2 764 810	0.8466886
Eriophorum vaginatum pine bogs	LC	3	2 536 176	0.8488997
ridge-hollow pine bogs	LC	3	1 636 626	0.8420008
tall-sedge pine fens	LC	3	2 687 747	0.8536454
flark pine fens	LC	3	1 939 429	0.8552710
open swamps	LC	3	940 430	0.8911394
Sphagnum papillosum fens	NT	6	1 916 682	0.8430770
Carex globularis pine mires	NT	6	1 852 450	0.8984205
thin-peated pine mires	NT	6	2 440 882	0.9019918
tall-sedge spruce-birch fens	NT	6	2 212 015	0.8675883
low-sedge pine fens	NT	6	2 603 590	0.8574391
rich flark fens	NT	6	830 654	0.9373695
birch swamps	NT	6	926 213	0.8857414
willow swamps	NT	6	734 389	0.8978095
herb rich sedge fens	VU	9	682 449	0.9275569
spruce-pine mires	VU	9	2 049 689	0.8742053
thin-peated spruce mires	VU	9	2 085 476	0.9068562
thin-peated rich spruce mires	VU	9	900 302	0.9244929
herb-rich spruce mires	VU	9	2 007 528	0.8897415
other dwarf shrub spruce mires	VU	9	2 104 097	0.8773428
rich spruce-birch fens	VU	9	558 369	0.9205659
Sphagnum papillosum pine fens	VU	9	1 108 069	0.8450825
rich pine fens	VU	9	1 312 442	0.9141347
rich spring fens	VU	9	877 313	0.9522184
rich birch fens	VU	9	284 666	0.9539643
Alnus glutinosa swamps	VU	9	134 056	0.8786442
Equisetum sylvaticum spruce mires	EN	12	1 333 126	0.9190212
Eriophorum vaginatum birch fens and *Carex nigra* birch fens	EN	12	1 073 341	0.8759565
rich swamp fens	EN	12	178 859	0.9455089
rich lawn fens	EN	12	427 740	0.9381893
Myrica gale swamps	EN	12	28 659	0.9210170
Alnus incana swamps	CR	15	39 746	0.9165840
Vascular plant and moss species				
LC, otherwise interesting species	-	1	1 002 638	1.0000000
NT, RT and DD species	-	2	2 257 702	1.0000000
CR, EN and VU species	-	3	2 148 028	1.0000000
Birds				
Emberiza pusilla	LC	1	419 949	1.0000000
Calidris falcinellus	LC	1	645 588	1.0000000
Lymnocryptes minimus	LC	1	803 685	1.0000000
Numenius arquata	LC	1	3 258 354	1.0000000
Numenius phaeopus	LC	1	2 763 938	1.0000000
Pluvialis apricaria	LC	1	2 995 380	1.0000000
Tringa erythropus	LC	1	897 799	1.0000000
Tringa glareola	LC	1	3 439 271	1.0000000
Tringa nebularia	LC	1	3 232 344	1.0000000
Anser fabalis	NT	2	1 657 656	1.0000000

Gavia stellata	NT	2	1 370 234	1.0000000
Lagopus lagopus	NT	2	2 413 535	1.0000000
Circus cyaneus	VU	3	1 244 000	1.0000000
Emberiza rustica	VU	3	2 728 321	1.0000000
Falco peregrinus	VU	3	1 368 552	1.0000000
Phalaropus lobatus	VU	3	305 567	1.0000000
Calidris pugnax	EN	4	1 100 836	1.0000000
Streams and ponds	-	1	109 477	1.0000000

Table S2. Each biodiversity feature's local weight in each administrative unit, and a feature's conservation status, if classified. Conservation status in south Finland covers zones 1–3 and status in north Finland zone 4. NA means that feature in question does not occur in a given area and a dash means that feature in question has not been evaluated or has not been evaluated separately for south and north Finland. Some of the names for the mire complexes do not have English equivalents.

Mire habitat complexes	1a + 1b Hemibor.	2a S bor.	2b S bor.	3a Middle bor.	3b Middle bor.	3c Middle bor.	Status in S Finland	4a N bor.	4b N bor.	Status in N Finland
Transition states between the mire complex forms	3	3	3	3	3	3	-	3	3	-
Not identified	3	3	3	3	3	3	-	3	3	-
plateau raised bogs	6	6	6	6	6	6	NT	6	6	NA
kalliopainannesuot	9	9	9	9	9	9	-	9	9	-
rantavalli- ja dyynikenttien suot	9	9	9	9	9	9	-	9	9	-
vesistönlaskusuot	9	9	9	9	9	9	-	9	9	-
pienet avosuot	9	9	9	9	9	9	-	9	9	-
northern boreal aapa mires	3	3	3	3	3	3	NA	3	3	LC
northern boreal sloping fens	3	3	3	3	3	3	NA	3	3	LC
luokituksen ulkopuoliset vaihettumat, streched	0	0	0	0	0	0	-	0	0	-
ei määritetty, streched	0	0	0	0	0	0	-	0	0	-
concentric raised bogs	6	6	6	6	6	6	NT	6	6	NA
plateau raised bogs, streched	0	0	0	0	0	0	NT	0	0	NA
eccentric raised bogs	9	9	9	9	9	9	VU	3	3	LC
Sphagnum fuscum raised bogs	9	9	9	9	9	9	VU	3	3	LC
middle boreal flark-surfaced aapa mires	9	9	9	9	9	9	VU	6	6	NT
middle boreal sloping fens	9	9	9	9	9	9	VU	9	9	NA
seasonal wetlands	9	9	9	9	9	9	DD	9	9	DD
harjunlievesuot	9	9	9	9	9	9	-	9	9	-
suppasuot	9	9	9	9	9	9	-	9	9	-
kumpumoreenialueisiin liittyvät suot	9	9	9	9	9	9	-	9	9	-
jokimeanderisuot	9	9	9	9	9	9	-	9	9	-
virtavesien rantasuot	9	9	9	9	9	9	-	9	9	-
lampien ja järvien rantasuot	9	9	9	9	9	9	-	9	9	-
jokiterassisuot, joki- ja purolaaksojen suot	9	9	9	9	9	9	-	9	9	-
rannikon ja saariston nuoret suot ja soiden kehityssarjat	9	9	9	9	9	9	-	9	9	-
puustoiset piensuot, erityisesti korvet	9	9	9	9	9	9	-	9	9	-
letot	9	9	9	9	9	9	-	9	9	-
lähteiköt, lähdesuot ja muut pohjavesivaikutteiset suot	9	9	9	9	9	9	-	9	9	-

paikalliset suoyhdistymät	9	9	9	9	9	9	-	9	9	-
suometsämosaiikit	9	9	9	9	9	9	-	9	9	-
kalliopainannesuot, streched	9	9	9	9	9	9	-	9	9	-
rantavalli- ja dyynikenttien suot, streched	9	9	9	9	9	9	-	9	9	-
vesistönlaskusuot, streched	9	9	9	9	9	9	-	9	9	-
pienet avosuot, streched	9	9	9	9	9	9	-	9	9	-
wooded raised bogs	12	12	12	12	12	12	EN	12	12	NA
middle boreal lawn-surfaced aapa mires	12	12	12	12	12	12	EN	12	12	NA
mire succession series of the land uplift coast	15	15	15	15	15	15	CR	15	15	NA
Mire habitats										
hollow bogs	6	6	6	6	6	6	NT	3	3	LC
flark fens	6	6	6	6	6	6	NT	3	3	LC
swamp fens	6	6	6	6	6	6	NT	3	3	LC
ombrotrophic low-sedge bogs	6	6	6	6	6	6	NT	3	3	LC
minerotrophic low-sedge fens	9	9	9	9	9	9	VU	3	3	LC
tall-sedge fens	9	9	9	9	9	9	VU	3	3	LC
Sphagnum fuscum bogs	3	3	3	3	3	3	LC	3	3	LC
dwarf shrub pine bogs	6	6	6	6	6	6	NT	3	3	LC
Eriophorum vaginatum pine bogs	6	6	6	6	6	6	NT	3	3	LC
ridge-hollow pine bogs	3	3	3	3	3	3	LC	3	3	LC
tall-sedge pine fens	9	9	9	9	9	9	VU	3	3	LC
flark pine fens	6	6	6	6	6	6	NT	3	3	LC
open swamps	6	6	6	6	6	6	NT	3	3	LC
Sphagnum papillosum fens	9	9	9	9	9	9	VU	3	3	LC
Carex globularis pine mires	9	9	9	9	9	9	VU	3	3	LC
thin-peated pine mires	6	6	6	6	6	6	NT	3	3	LC
tall-sedge spruce-birch fens	9	9	9	9	9	9	VU	6	6	NT
low-sedge pine fens	9	9	9	9	9	9	VU	6	6	NT
rich flark fens	15	15	15	15	15	15	CR	6	6	NT
birch swamps	9	9	9	9	9	9	VU	3	3	LC
willow swamps	6	6	6	6	6	6	NT	3	3	LC
herb rich sedge fens	15	15	15	15	15	15	CR	6	6	NT
spruce-pine mires	9	9	9	9	9	9	VU	6	6	NT
thin-peated spruce mires	9	9	9	9	9	9	VU	3	3	LC
thin-peated rich spruce mires	12	12	12	12	12	12	EN	6	6	NT
herb-rich spruce mires	12	12	12	12	12	12	EN	6	6	NT
other dwarf shrub spruce mires	9	9	9	9	9	9	VU	6	6	NT
rich spruce-birch fens	15	15	15	15	15	15	CR	9	9	VU
Sphagnum papillosum pine fens	9	9	9	9	9	9	VU	6	6	NT
rich pine fens	15	15	15	15	15	15	CR	9	9	VU
rich spring fens	15	15	15	15	15	15	CR	6	6	NT
rich birch fens	15	15	15	15	15	15	CR	6	6	NT
Alnus glutinosa swamps	9	9	9	9	9	9	VU	9	9	NA

Equisetum sylvaticum spruce mires	12	12	12	12	12	12	EN	9	9	VU
Eriophorum vaginatum birch fens and *Carex nigra* birch fens	12	12	12	12	12	12	EN/EN	6	6	NT/NA
rich swamp fens	15	15	15	15	15	15	CR	12	12	EN
rich lawn fens	15	15	15	15	15	15	CR	12	12	EN
Myrica gale swamps	12	12	12	12	12	12	EN	12	12	NA
Alnus incana swamps	15	15	15	15	15	15	CR	15	15	NA
Plant and moss species										
LC and otherwise interesting species	1	1	1	1	1	1	-	1	1	-
NT, RT and DD species	2	2	2	2	2	2	-	2	2	-
CR, EN and VU species	3	3	3	3	3	3	-	3	3	-
Birds										
Emberiza pusilla	1	1	1	2	1	1	-	1	1	-
Calidris falcinellus	1	1	1	2	2	2	-	1	1	-
Lymnocryptes minimus	1	2	2	2	1	1	-	1	1	-
Numenius arquata	2	1	1	1	1	1	-	1	1	-
Numenius phaeopus	1	2	2	1	1	1	-	1	1	-
Pluvialis apricaria	1	2	2	1	1	1	-	1	1	-
Tringa erythropus	1	1	1	2	2	1	-	1	1	-
Tringa glareola	2	2	2	1	1	1	-	1	1	-
Tringa nebularia	1	2	1	1	1	1	-	1	1	-
Anser fabalis	2	2	2	2	2	2	-	2	2	-
Gavia stellata	2	2	2	2	2	2	-	2	2	-
Lagopus lagopus	2	2	2	2	2	2	-	2	2	-
Circus cyaneus	3	3	3	3	3	3	-	3	3	-
Emberiza rustica	3	3	3	3	3	3	-	3	3	-
Falco peregrinus	3	3	3	3	3	3	-	3	3	-
Phalaropus lobatus	3	3	3	3	3	3	-	3	3	-
Calidris pugnax	4	4	4	4	4	4	-	4	4	-
Streams and ponds	2	2	2	2	2	2	-	1	1	-

Table S3. Number of cells of each forest vegetation zone and zone-specific weight calculated by dividing number of cells by total number of cells.

Forest vegetation zone	Number of cells	Area in hectares	Weight
1a and 1b Hemiboreal	27 789	6 947	0.00748
2a Southern boreal	289 392	72 348	0.07788
2b Southern boreal	240 993	60 248	0.06485
3a Middle boreal	1 569 082	392 271	0.42226
3b Middle boreal	665 969	166 492	0.17922
3c Middle boreal	205 276	51 319	0.05524
4a Northern boreal	379 670	94 918	0.10217
4b Northern boreal	337 742	84 436	0.09089
Total	3 715 913	928 979	1.0

Some of the considered biodiversity features include significant south to north variation in their rarity and consequently have strongly differing habitat Red List statutes for northern and southern parts of Finland. For example, rich fens are among the most threatened habitats in Southern Finland, but without considering their local rarity in the south, their relatively large northern abundance would have been resulted in lowered rarity-based priority for them throughout the country. To be able to consider both national and local scale rarity and threatedness of the biodiversity features, the analysis was executed in a way, where national priorities were balanced with local priorities using the administrative units approach (ADMU) (Moilanen and Arponen 2011). The used method calculates the irreplaceability of areas both locally (here forest vegetation zones) and globally (here nationally) and combines this information. The balance between local and global priorities can be adjusted and the analysis iterated accordingly. Consideration of local irreplaceability through the administrative units also leads to a more balanced division of protected areas among the regions. This kind of a more balanced spatial coverage of the protected area network in Finland was also supporting the goals of the CMPP.

The planning area was divided into 9 regions or administrative units according to forest vegetation zones. Using the method required us to calculate relative weights for each administrative unit by dividing each unit’s number of cells by the total number of all units’ cells (Table S3). The weights were used to ensure a balanced solution over the whole planning area (Moilanen et al. 2014). In addition to the national-scale weights of biodiversity features, we gave regional weights for

biodiversity features in each administrative unit according to their regional Red List statuses (Table S1). In the case of features unclassified in the habitat Red List, we used an expert opinion. We verified the balance between regional and national scale rarity of features by iterating the analysis and checking the biodiversity features' performance curves, which showed that local rarity was affected as planned without overly compromising the national scale performance (i.e. emphasis on nationally threatened habitats and species).

Hierarchical masks

When prioritizing *Obligatory* and *Balancing scenarios*, we used a two-phase mask that removed first all the candidate mires and then the existing protected mires (for more information about masks, see e.g. Kremen et al. 2008; Moilanen et al. 2014). When prioritizing *Voluntary scenario*, we used a three-phase hierarchical mask to force the exclusion of the mires where even a single landowner resisted protection. The mask determined the removal order of the mires: first the resistance-free candidate mires, then the candidate mires having resistance, and finally the existing protected mires.

Effect of resistance in Balancing scenario

When prioritizing *Balancing scenario*, we included landowners' resistance to protection as a feature layer, where the cell values were set as zero in the existing protected mires and between 0 and 1 in the candidate mires, according to their resistance proportion. To make the prioritization avoid resisted mires, we set a minus weight for the resistance layer (Moilanen et al. 2011). We iterated the analysis with weights of 0, –20, –50 and –100 for the resistance to investigate the relative differences of different weights and resulting trade-offs. Prioritization curves for resistance showed that using the weight of –100 would have been almost equivalent to the total exclusion of all resisted mires (Fig. S2). Instead, the weight of –50 effectively excluded resisted mires from the top fraction, still allowing valuable biodiversity features located in resisted mires to prevent all the

exclusions. On the other hand, biodiversity representation produced by the weight of –20 differed only little from that produced by the weight of –50, but caused almost double resistance. Therefore, we chose the weight of –50 to serve as a minus weight for the resistance layer in *Balancing scenario*.

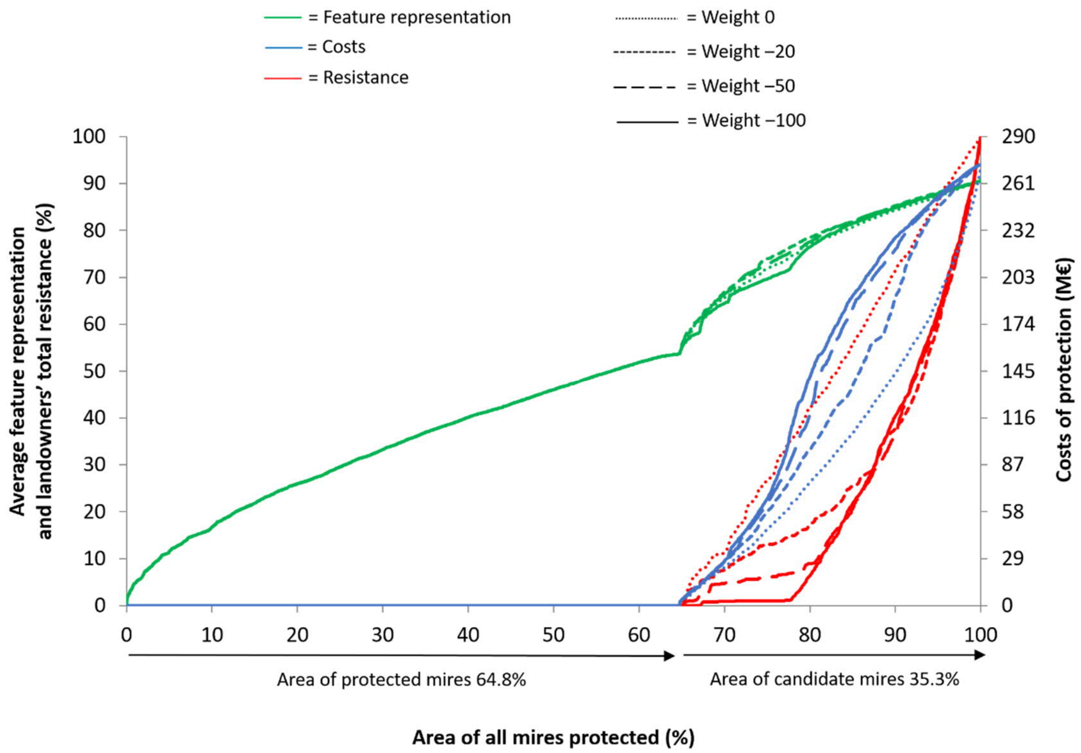

Figure S2. Average representation of biodiversity features (green curve), landowners' total resistance (red curve), and costs of conservation (blue curve) for each prioritization testing the effect of different minus weights of resistance to protection. Small dotted lines represent zero weighting, medium dotted lines weight of –20, dash lines weight of –50, and solid lines weight of –100.

References

Kareksela, S., K. Aapala, A. Alanen, T. Haapalehto, J. S. Kotiaho, J. Lehtomäki, N. Leikola, N. Mikkonen, et al. 2020. Combining spatial prioritization and expert knowledge facilitates effectiveness of large-scale mire protection process in Finland. *Biological Conservation* 241. doi:10.1016/j.biocon.2019.108324.

Kremen, C., Cameron, A., Moilanen, A., Phillips, S.J., Thomas, C.D., Beentje, H., Dransfield, J., Fisher, B.L. et al. 2008. Aligning Conservation Priorities Across Taxa in Madagascar with High-Resolution Planning Tools. *Science* 320:222–226. doi:10.1126/science.1155193

Moilanen, A., Anderson, B.J., Eigenbrod, F., Heinemeyer, A., Roy, D.B., Gillings, S., Armsworth, P.R., Gaston, K.J. et al. 2011. Balancing alternative land uses in conservation prioritization. *Ecological Applications* 21: 1419–1426. https://doi.org/10.1890/10-1865.1

Moilanen, A., and Arponen, A. 2011. Administrative regions in conservation: Balancing local priorities with regional to global preferences in spatial planning. *Biological Conservation* 144, 1719–1725. https://doi.org/10.1016/j.biocon.2011.03.007

Moilanen, A., Pouzols, F.M., Meller, L., Veach, V., Arponen, A., Leppänen, J., and Kujala, H. 2014. Zonation - Spatial conservation planning methods and software. Version 4. User Manual. https://github.com/cbig/zonation-core/releases

Rassi, P., Hyvärinen, E., Juslen, A., and Mannerkoski, I. 2010. The 2010 Red List of Finnish Species (in Finnish, English summary). Available in http://hdl.handle.net/10138/299501

Raunio, A., Schulman, A., and Kontula, T. 2008. Assessment of threatened habitat types in Finland (in Finnish, English summary). Available in http://hdl.handle.net/10138/37900

Appendix S4

Figure S3

See below.

a)

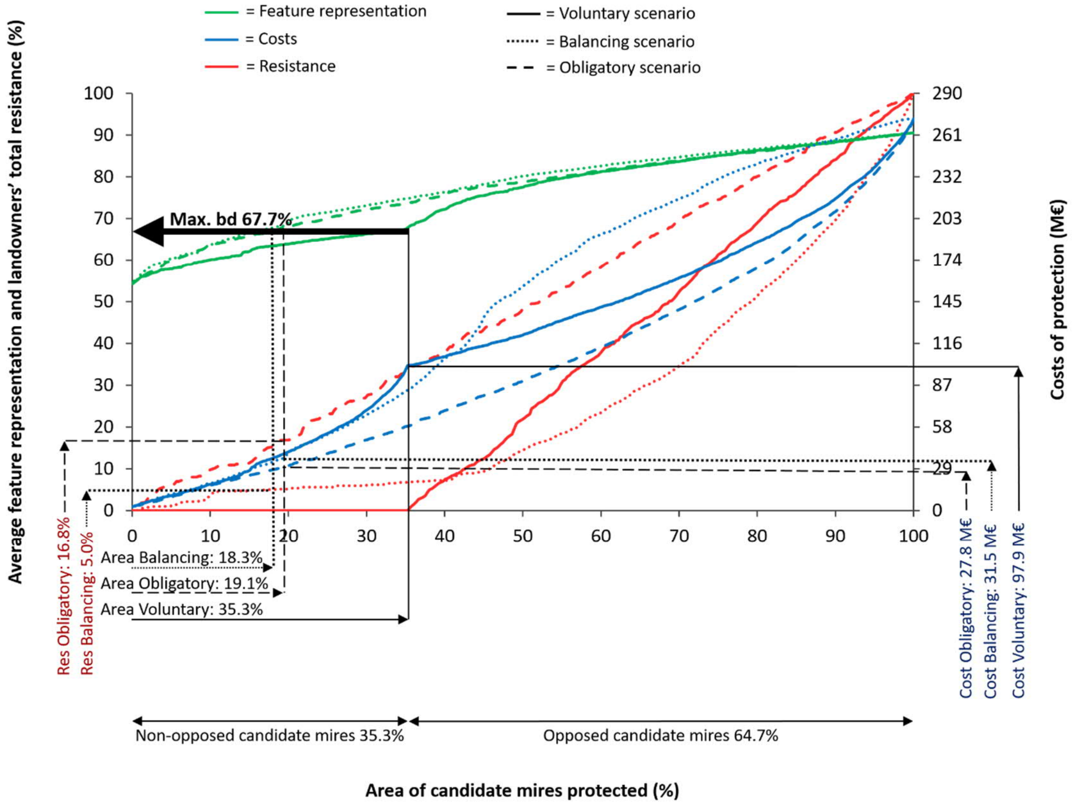
= Feature representation
= Costs
= Resistance
= Voluntary scenario
= Balancing scenario
= Obligatory scenario
Max. bd 67.7%
Average feature representation and landowners' total resistance (%)
Costs of protection (M€)
Res Obligatory: 16.8%
Res Balancing: 5.0%
Area Balancing: 18.3%
Area Obligatory: 19.1%
Area Voluntary: 35.3%
Cost Obligatory: 27.8 M€
Cost Balancing: 31.5 M€
Cost Voluntary: 97.9 M€
Non-opposed candidate mires 35.3%
Opposed candidate mires 64.7%
Area of candidate mires protected (%)

b)

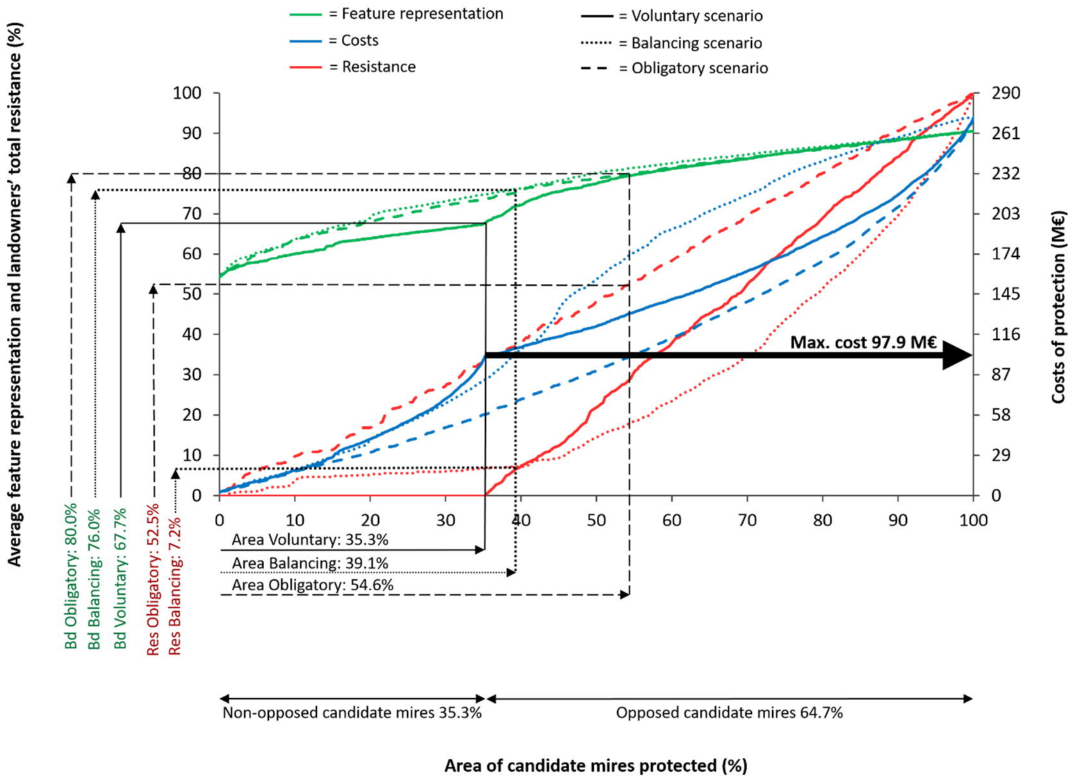
= Feature representation
= Costs
= Resistance
= Voluntary scenario
= Balancing scenario
= Obligatory scenario
Max. cost 97.9 M€
Average feature representation and landowners' total resistance (%)
Costs of protection (M€)
Bd Obligatory: 80.0%
Bd Balancing: 76.0%
Bd Voluntary: 67.7%
Res Obligatory: 52.5%
Res Balancing: 7.2%
Area Voluntary: 35.3%
Area Balancing: 39.1%
Area Obligatory: 54.6%
Non-opposed candidate mires 35.3%
Opposed candidate mires 64.7%
Area of candidate mires protected (%)

c)

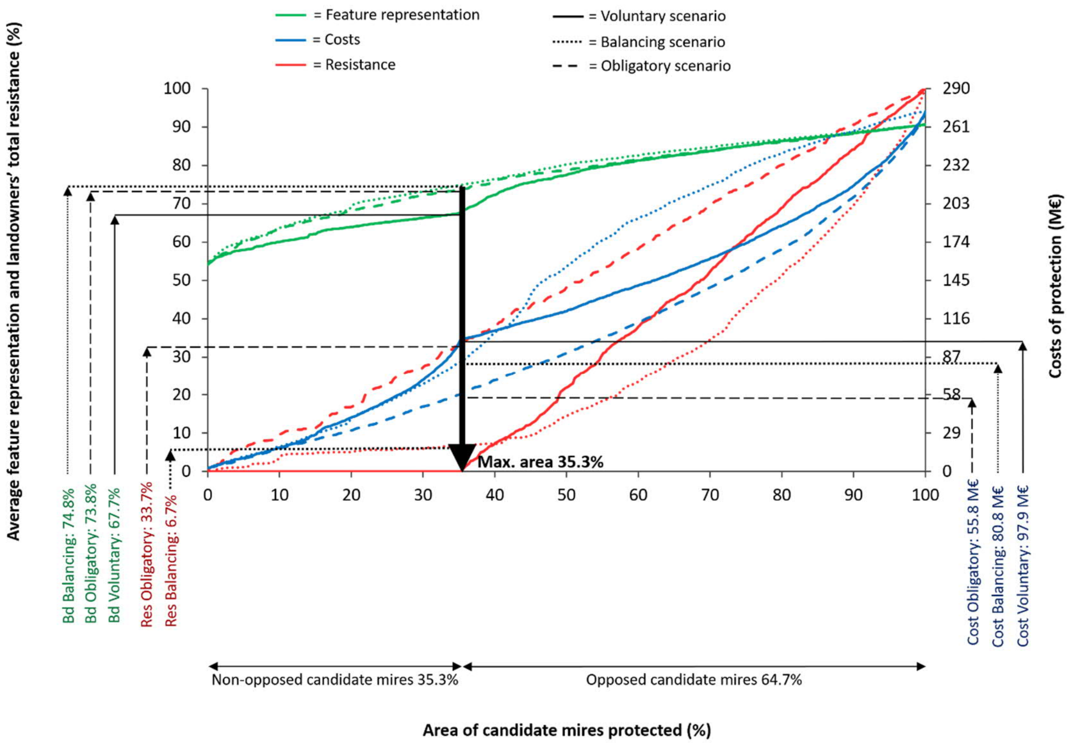

Figure S3. Average representation of biodiversity features (%) (green curves), landowners' resistance to protection (%) (red curves), and costs of protection (million euros) (blue curves) for each conservation scenario. Thick black arrows point out the values produced by *Voluntary scenario* against which other solutions are compared to. Black solid lines and arrows represent *Voluntary scenario*, black dotted lines and arrows *Balancing scenario*, and black dashed lines and arrows *Obligatory scenario*. a) Feature representation protected is that of *Voluntary scenario*, i.e. 67.7%. b) Costs for protection are those of *Voluntary scenario*, i.e. 97.9 million euros. c) Area for protection is that of *Voluntary scenario*, i.e. 35.3% of the candidate mires' area. Average feature representation does not reach 100% because the included mire complexes and habitats suffer from decreased condition caused by drainage, expressed by the curves.
